# Feeling of Meaningfulness and Anxiety of Taekwon-Do Fighters in a Salutogenic Notion

**DOI:** 10.3390/ijerph192315658

**Published:** 2022-11-25

**Authors:** Dorota Ortenburger, Dariusz Mosler, Józef Langfort, Jacek Wąsik

**Affiliations:** Department of Kinesiology and Health Prevention, Jan Dlugosz University of Czestochowa, 42-200 Czestochowa, Poland

**Keywords:** taekwon-do, meaningfulness, anxiety, sense of coherence, mental health, well-being

## Abstract

This study aimed to examine the relation between the feeling of meaningfulness and also the characteristics of engaged participation (namely, the frequency of participation in voluntary groupings) and the level of anxiety among those who train a group of elite taekwon-do fighters. The research encompassed 58 people, all of whom were taekwon-do ITF (International Taekwon-do Federation) athletes at an elite level. The Questionnaire of Life Orientation (SOC-29) and the Inventory of the State and Features of Anxiety were used. The data were supplemented by the authors’ own questions referring to activities in the field of taekwon-do. The group of taekwon-do fighters chosen was internally divided with regard to the level of the state of anxiety and the feeling of meaningfulness (*p* < 0.01). It was found that, together with the growth in the values stipulated in the accepted model, the frequency of taekwon-do groupings (β = −0.38), as well as the feeling of meaningfulness (β = −0.31), the value of the level of intensification of the state of anxiety dropped. The data revealed that, together with age, the level of anxiety decreased and the feeling of meaningfulness increased. The difference in the levels of anxiety between women and men was statistically non-significant (*p >* 0.05). The research findings illustrate that the feeling of meaningfulness and participation in groupings constitute a differentiating factor in terms of the intensification of the average level of anxiety in the elite taekwon-do group. More frequent participation in training goes hand-in-hand with the greater feeling of meaningfulness; perhaps, this is associated with the specific training, which, among other factors, favours adaptation to challenges and actions under pressure.

## 1. Introduction

Acquiring proficiency in sports competitions is associated with, among other factors, the systematic undertaking of physical effort, while also involving intricate skills and predisposition [1], as well as the psychological feeling of the purposefulness of the effort made. This is directly connected with the understanding of the justified ties between the situations or events and personal action, which we shall describe here as the feeling of meaningfulness. It fulfils a significant role in shaping the relations between a situation, stress (including anxiety), an individual response, and the adjustable mechanisms of the entity [2]; simultaneously, this notion is consistent with the concept of the intricate approach to the issue of a healthy lifestyle [3] and is treated as one of the predictors of mental health [4,5].

The feeling of meaningfulness in the salutogenic sense constitutes one of the components of the feeling of coherence. It is a type of generalized life orientation, whereby there is a clear conviction that life has sense and order; this signifies that the occurring events are predictable. The requirements of life are challenges that are worth the effort and engagement, while also being possible to fulfil [6]. This concept assumes that maintaining and regaining balance requires a person to constantly activate the features of the entity that favour the continuous movement in the direction of balance, harmony, and health.

Scientific research has been carried out among those training in karate in terms of the feeling of meaningfulness and the remaining two composite elements of the feeling of coherence [7]. This research confirmed their participation in the motivation and realization of intentions. The feeling of understandability and sense grew with age, and they were positively correlated with the feelings of ties with nature. Understanding both the natural environment as well as the usefulness of establishing sporting goals co-existed with the motivation to increase efforts in training. The stronger feeling of meaningfulness was probably associated with the possibility of a clearer perception of the significance of current events that were transient from a broader perspective. In a certain way, this may have favoured resilience in terms of the execution of long-term goals [8]. The research conducted on taekwon-do illustrates that the increased skills of coping with frustration, as well as the emotions associated with anger, are favourable to health [9].

The feeling of meaningfulness is acknowledged to be a factor that has a favourable impact on the state of health, while also improving the psychophysical and physical state of an individual and, possibly, also enhancing resistance to stressful events in life, including stress itself. This constitutes part of the general life orientation and has an impact on the proactive strategies of coping with fear or anger [10]. More adaptive strategies of coping with stress were found among nurses with a stronger feeling of coherence than amongst those with an average or low level of this factor. Anxiety and other stressful situations may be perceived as challenges, not only as threats [7]. Research shows that with the aim of enhancing efficiency in terms of professional activity, interventions are conducted in order to increase the level of meaningfulness [11,12,13].

The feeling of meaningfulness may be a factor that favors development on the road to self-development. In the field of martial arts, this notion is consistent with the claim that systematic training of taekwon-do favours concentrating on the process of striving towards the goal [1]. Additional forms in the manner of taekwon-do groupings and camps constitute an intensified form of this activity that requires physical effort [14]; however, not everyone is strongly involved in the achievement of a goal, the more so as they find themselves in new or surprising situations.

Hence, this study aimed to examine the relation between the feeling of meaningfulness and also the characteristics of engaged participation (namely, the frequency of participation in voluntary groupings) and the level of anxiety among people conducting taekwon-do training.

Questions are proposed such as asking how these relations are formed and in what way possible differences are manifested in the field of these factors amongst taekwon-do fighters.

## 2. Materials and Methods

### 2.1. Subject

The research encompassed a total of 58 people, comprising 32 men and 26 women (age: 27.67 ± 9.14; range: from 18 to 45 years of age). All of them were taekwon-do ITF (International Taekwon-do Federation) athletes at an elite level (1 cup to 2 dan belt) with average training experience of 10.67 years (minimum 5 and maximum 26 years of training and competition). Inclusion criteria: at least 5 years of training, at least 1 cup, participation in sports competitions (regardless of the type of competition: formal systems, combat, etc.), training at least four times a week of 1.5 h.

All participants were informed of the testing procedures and signed informed consents for voluntary participation in this study. The study was approved by the Ethics Committee of the University in Rzeszow (number 2/6/2017, date 8 June 2017).

### 2.2. Protocol

The gauging was conducted by means of standardized tools in the field of health psychology such as the following:

(1) The Scale of Meaningfulness as an element of the Questionnaire of Life Orientation (SOC-29) for the purpose of analysing the feeling of coherence [6]. An analysed person gave answers with the aid of a 7-degree Likert scale, whereby 1 signifies that the given attitude exists on a permanent basis, while 7 signifies never. The general score was calculated, namely, the intensity of the feeling of meaningfulness of coherence among those analyzed (the result was in a range of 8–56);
(2) The Inventory of the State and Features of Anxiety was applied, which is based on the differentiation between anxiety perceived as transitory and situational in terms of the state of the entity, while anxiety is perceived as a relatively constant feature of a personality. In our research, we concentrated on the state of anxiety as it is associated with anxiety that is conditioned situationally [15,16], which occurs, among other cases, during sporting rivalry in taekwon-do [17];
(3) The data were supplemented by the authors’ questions with regard to activity in the field of taekwon-do.

### 2.3. Statistics

Basing our work on the available literary sources [18,19], in order to analyze the responses we conducted an analysis of the k-average cluster by recommending a 2-cluster solution. All the clusters were identified in our set of data as follows: meaningfulness, and state of anxiety.

We adopted the model of statistical analysis with caution, in terms of the quantity of data in our possession, which facilitated familiarization with the dependencies, namely, k-average (k-means clustering). Thanks to this fact, it became possible to acquire the result and information identified by our team in the research conducted on these concentrations (clusters) relating to how they differ from each other in terms of each dimension formulated in the subject matter of this paper (in terms of variables: the generalized feeling of meaningfulness and state of anxiety).

For all the registered indicators, the average and standard deviations were designated. In terms of the description as to how the general trend of coefficients between the variables was formed, an analysis of the regression was applied. The correlation between the chosen indicators was designated. Analysis of the ANOVA variance was conducted.

Taking account of the number of cases, a choice was reached in terms of the number of variables by adhering to the guidelines in the sphere of statistics [20] which recommend analysing a minimum of 10 times more cases than the variables that exist in the model of stepwise regression.

The statistical significance was accepted at the level of *p* < 0.05. All calculations were made with the aid of Statistica 12.00 (StatSoft Europe, Hamburg, Germany).

## 3. Results

Table 1 contains descriptive statistics of variables: levels of anxiety and the feeling of meaningfulness. The statistics associated with resolving the two clusters are presented in Table 2. The parameters of distribution of the clusters’ differentiation in terms of the levels of anxiety and the feeling of meaningfulness were stipulated. Statistical analysis of the data revealed that a group of the analysed taekwon-do fighters is internally divided with regard to the state of anxiety and the feeling of meaningfulness (*p* < 0.01).

The research findings based on the model of stepwise regression under analysis indicated in Table 3 illustrate that the growth of values stipulated in the accepted model of frequency of taekwon-do groupings (β = −0.38), and also the feeling of meaningfulness (β = −0.31) of the level of the state of anxiety, drops. In the first iteration, those two predictors explain 46% of the variables of the state of anxiety; thus, over half of them remain unexplained; yet, the automated adjustment ignored this step as components were not statistically significant. Rejecting meaningfulness left statistically significant variables regarding participation, with similar exploratory power of 46% of cases.

**Figure 1:**
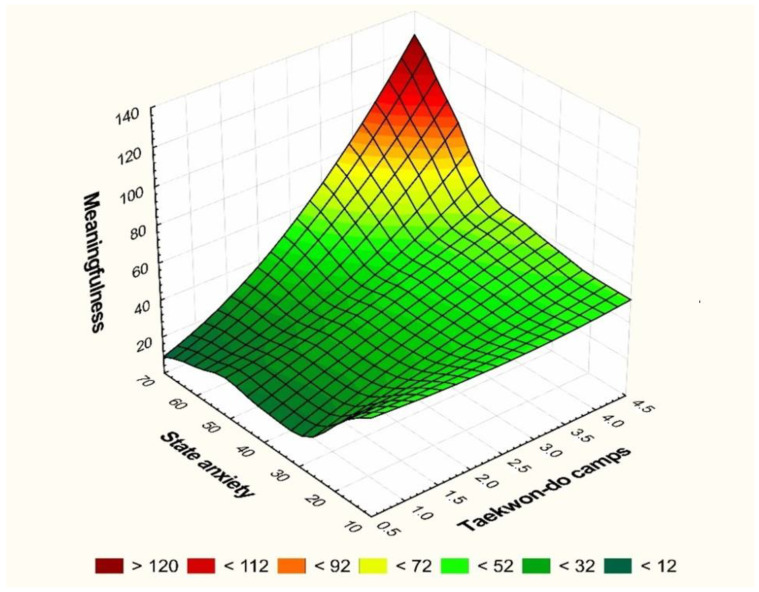
Dependencies between the level of the state of anxiety and the level of meaningfulness and frequency of taekwon-do camps.

It is possible to note that a greater participation in camps is consistent with a higher level of the feeling of meaningfulness and a lower intensity of anxiety. There was moderate correlation between age and the level of intensity of anxiety (r = 0.48) and weak correlation between age and the feeling of meaningfulness (r = 0.38). The data presented in Figure 1 reveal that, together with attendance at taekwon-do camps, the level of anxiety decreases, while the feeling of meaningfulness increases.

**Figure 2:**
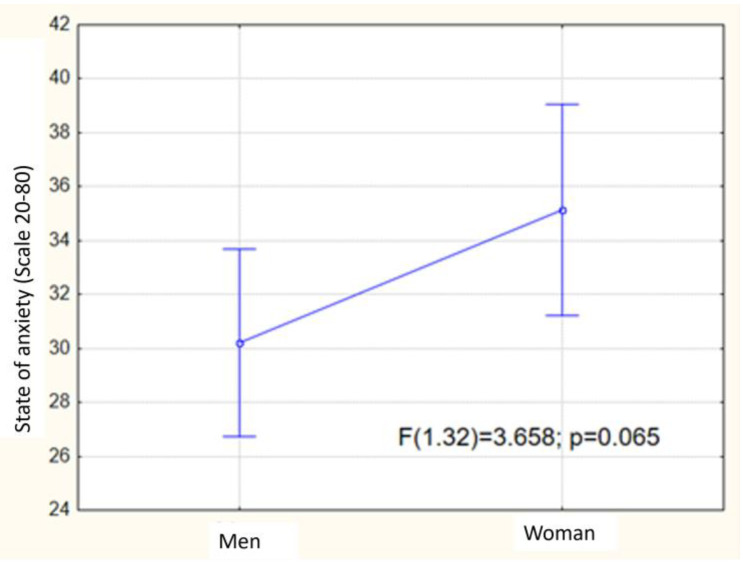
Graphic interpretation of the state of anxiety with regard to sex type (non-significant *p* > 0.05).

In Figure 2, a graphic interpretation of the level of anxiety (state of anxiety) has been presented with regard to sex type. It can be seen that the difference in the level of anxiety between women and men is minor, does not exceed 4 points on average, and is statistically non-significant (*p* = 0.065).

## 4. Discussion

The analysis conducted illustrates that the feeling of meaningfulness differs in terms of the intensity of active participation of fighters at taekwon-do camps. Taking into account the fact that in this form of martial arts there is the internalization of moral principles, these results are understandable [21,22]. Metanalysis existing in the literature stating that taekwon-do correlates with sociality, character, etiquette, and school life adjustment may be helpful for future studies to include the variables, measure, and results of that paper.

It is also possible to interpret this from the perspective of the aims that are executed during the course of cyclical sports groupings [23,24]. There is also research which illustrates that the clear aims of groupings and camps favour the feeling of responsibility for the activities associated with training, the rhythm of the day, and nutrition [14,22,25,26]. More frequent participation in taekwon-do activities is consistent with a lower level of anxiety. Together with the reduction of anxiety, there may also be an increase in the possibilities of resolving a particular problem in a flexible way. The motivation to commence various purposeful activities and to continue despite the obstacles grows. Regular participation in taekwon-do training is associated with the mechanisms responsible for changing behavior in such a way as to constitute a normal, internalized, everyday element in the life activity of an individual; additionally, the analysis conducted reveals that the frequency of training during the week was covariant with the feeling of meaningfulness (r = 0.69; *p* < 0.01). Gaining effectiveness in combat sports is associated with not only the systematic use of physical effort, but also with coping with stress. Such actions are undertaken with a specific goal in mind and have significance that is strictly associated with the development of skills and experience in fights, whereby the fighters have the sense of meaningfulness of the actions undertaken by them and face situations and their own psycho–physical reactions; thus, it was accepted in our research that the assumption that a high level of state of anxiety is felt by the analyzed fighters as a reaction to stressful events and not trait anxiety as state of anxiety is usually an adaptive reaction and finishes once the danger has passed.

Our research corresponds in this field with the reports relating to state of anxiety among taekwon-do fighters [17]. As the regression model explains only 46% of cases, we could assume that active participation and sense of meaningfulness have significant impact in shaping the state of anxiety in some individuals, but its exploratory power is not a sufficient factor in explaining the variability completely; therefore, further discussion is focused on sense of purpose and meaningfulness, as clustering analysis in Table 2 reveals more straight division and presents more straightforward relation between the presented variables.

Furthermore, frequent participation in training and camps associated with this activity is an expression of readiness, and also the possibility of constant self-improvement, the development of new competences, and also flexible reactions to various and constantly changing situations. The feeling of meaningfulness may support the long-term effort of demanding activities undertaken during the course of personal development.

Our research indicates that the feeling of anxiety reduces with age (r = −0.48; *p* < 0.05), while there is a growth in the feeling of meaningfulness (r = 0.39; *p* < 0.05) amongst those training in taekwon-do; perhaps, this is the result of, among other factors, the development of new strategies of coping with pressure and difficult situations and an increase in the feeling of self-confidence, which taekwon-do requires [27,28,29].

Research and reports in the literature show that there is a clear difference in terms of the frequency of anxiety disorders among women and men [30,31,32,33]. Our research reveals that in accordance with the expectations, the level of anxiety amongst women is greater than among men, albeit this difference does not illustrate a statistical significance (Figure 2). It indicates that martial arts training could limit gender differences in perceived anxiety levels. Women who engage in interaction, e.g., in the form of sparring, undertake activity that goes beyond their comfort zone where they feel safe; thus, they may adapt to a specified level of anxiety as a result of the feeling of meaningfulness of their actions [2]. Those analyzed show that taekwon-do fighters do not withdraw from participation in fights due to fears of difficulty, but, rather, apply proactive strategies of coping with stress to the highest degree as follows: revaluation and turning attention away from negative feelings [34]; hence, there are substantive reasons to think that the feeling of meaningfulness as an element of the generalized feeling of coherence is one of the important reasons behind a more effective way of coping with difficult situations. Our results enhance a view that martial arts could help people who need a sense of meaningfulness. Finding a purpose of life through group sport activities with controlled competitive aspects could help produce better social functioning in everyday life [23,35]. It is important to make sense of victory, but also to understand and make sense of defeat [36].

The data which we collected facilitated a more profound perception of the issue and the formulation of further hypotheses that require further verification. One of the limitations presented here is that of the number of people analyzed. This was influenced by the elite nature of the analyzed group and its specifics; regarding this, we feel that this may constitute the unique nature of the results acquired. This leads to an increase in the amount of knowledge that is useful in terms of the research conducted in the salutogenic model in the field of science relating to physical culture. This information also constitutes an introduction to a more profound analysis used in preparing strategies, training, and therapeutic programs. The research presented was first and foremost of an exploratory nature; simultaneously, we hope that it will lead to the development of knowledge on the issue of the possibility of the useful application of knowledge in terms of the role of the feeling of meaningfulness with regard to health and the harmonious, sustainable development of the lifestyle of an individual.

## 5. Conclusions

The feeling of meaningfulness and participation in groupings constitute a factor in terms of the differentiating intensities of the level of anxiety. Active and frequent participation in group forms of sport activities and training is consistent with a greater feeling of meaningfulness among taekwon-do participants.

Age and biological maturity only partially explain a level of anxiety and sense of meaningfulness of taekwon-do fighters, indicating that experience level and maturity in terms of physical fitness and education play important roles in handling one’s own mental health.

In the presented study, the average level of anxiety created by circumstances relating to women and men is not statistically significant; this allows us to recommend martial arts as a group form of physical activity regardless of the gender of the participants.

Further studies in a larger group are planned to solve the main problem. In future research, we will expand the research field to possible effects of chronological age, contextual factors, and social desirability on the obtained findings and differences between participants of technical and fight disciplines.

## Figures and Tables

**Table 1:** Descriptive statistic of variables.

Variables		Mean	Minimum	Maximum	SD
State of anxiety	Men	41.12	21.00	58.00	10.30
	Female	41.31	21.00	68.00	15.09
	Total	41.22	21.00	68.00	13.05
Meaningfulness	Men	36.50	14.00	56.00	12.95
	Female	33.54	14.00	49.00	10.46
	Total	35.17	14.00	56.00	11.89

SD—standard deviation.

**Table 2:** Parameters of distribution of clusters’ differentiation in terms of level of anxiety and feeling of meaningfulness (*p* < 0.01).

Variables	Cluster 1 Mean ± SD	Cluster 2 Mean ± SD	SSB	SSW	F	*p*
Meaningfulness	24.54 ± 7.57	42.67 ± 7.99	4626.87	3429.39	75.554	0.000
State of anxiety	53.75 ± 7.53	32.38 ± 7.46	6423.55	3284.53	109.519	0.000

SSB—Sum of squares between cluster; SSW—Sum of squares within clusters.

**Table 3:** Multiple regression models (stepwise regression) of state of anxiety level in the context of selected predictor variables.

Type	Iteration	Regression Model	Predictors	β	SE	*p*
State of anxiety	First step	R = 0.68; F(2.55) = 23.48 *p* < 0.000; R^2^ = 0.46 SE estimation = 9.76	Participation in TKD groupings	−0.388	0.215	0.076
			Feeling of meaningfulness of events	−0.310	0.214	0.153
	Second step	R = 0.66; F(1.56) = 44.06 *p* < 0.000; R^2^ = 0.46.01 SE estimation = 9.85	Participation in TKD groupings	−0.66	0.10	0.000

β—Unstandardized coefficient (obtained after running a regression model on variables measured in their original scales), SE—standard error β coefficient.

## Data Availability

Not applicable.
